# Influence of Cigarettes and Alcohol on the Severity and Death of COVID-19: A Multicenter Retrospective Study in Wuhan, China

**DOI:** 10.3389/fphys.2020.588553

**Published:** 2020-12-09

**Authors:** Mengyuan Dai, Liyuan Tao, Zhen Chen, Zhi Tian, Xiaofang Guo, Diane S. Allen-Gipson, Ruirong Tan, Rui Li, Li Chai, Fen Ai, Miao Liu

**Affiliations:** ^1^Department of Gynecological Oncology, Zhongnan Hospital of Wuhan University, Wuhan, China; ^2^Hubei Key Laboratory of Tumor Biological Behaviors, Wuhan, China; ^3^Hubei Cancer Clinical Study Center, Wuhan, China; ^4^Research Center of Clinical Epidemiology, Peking University Third Hospital, Beijing, China; ^5^Department of Emergency, The Central Hospital of Wuhan Affiliated to Tongji Medical College, Huazhong University of Science and Technology, Wuhan, China; ^6^Department of Pharmaceutical Sciences, Taneja College of Pharmacy, University of South Florida, Tampa, FL, United States; ^7^Department of Obstetrics & Gynecology, Morsani College of Medicine, University of South Florida, Tampa, FL, United States; ^8^Division of Allergy and Immunology, Department of Internal Medicine, College of Medicine, University of South Florida, Tampa, FL, United States; ^9^Department of Urology, Boston Children’s Hospital, Harvard Medical School, Boston, MA, United States; ^10^Department of Radiation Oncology, Sichuan Cancer Center, School of Medicine, Sichuan Cancer Hospital and Institute, University of Electronic Science and Technology of China, Chengdu, China; ^11^Department of Pathology, Brigham and Women’s Hospital, Harvard Medical School, Boston, MA, United States

**Keywords:** cigarette, alcohol, COVID-19, SARS-CoV-2, severity, death

## Abstract

**Background:**

The recent emergence and rapid global spread of coronavirus disease 2019 (COVID-19) is leading to public health crises worldwide. Alcohol consumption and cigarette smoking (CS) are two known risk factors in many diseases including respiratory infections.

**Methods:**

We performed a multi-center study in the four largest hospitals designated for COVID-19 patients in Wuhan. There are totally 1547 patients diagnosed with COVID-19 enrolled in the study, alcohol consumption and CS history were evaluated among these patients. The epidemiology, laboratory findings and outcomes of patients contracted COVID-19 were further studied.

**Results:**

Our findings indicated that COVID-19 patients with a history of CS tend to have more severe outcomes than non-smoking patients. However, alcohol consumption did not reveal significant effects on neither development of severe illness nor death rates in COVID-19 patients.

**Conclusion:**

CS is a risk factor for developing severe illness and increasing mortality during the SARS-CoV-2 infection. We believe that our findings will provide a better understanding on the effects of alcohol intake and CS exposure in COVID-19 patients.

## Introduction

The recent emergence and rapid global spread of Severe Acute Respiratory Syndrome (SARS) Coronavirus 2 (SARS-CoV-2) and the resulting coronavirus disease 2019 (COVID-19) is associated with more than 12,077,210 cases and more than 550,327 deaths worldwide as of July 9, 2020 (COVID-19 Map from Johns Hopkins Coronavirus Resource Center). Considering the high rate of SARS-CoV-2 transmission and that there is no known cure for this disease at present, identifying vulnerable populations will be crucial for taking measures to protect those who are at increased risk of infection or of severe disease from COVID-19 (Clark et al., 2020).

Alcohol consumption and cigarette smoking (CS) are two known risk factors in many diseases including respiratory infections (Traphagen et al., 2015; Han et al., 2019). Chronic alcohol consumption has been identified as an important risk factor for the development of acute respiratory distress syndrome (ARDS) (Moss et al., 2003), which is one of the most severe complications of COVID-19. CS has been well-recognized as a high-risk factor for respiratory diseases; however, there is still no evidence indicating that it increases the risk of SARS-CoV-2 infection. Even though there are numerous studies focused on the link between smoking and COVID-19, it is still unclear whether CS increases the severity of COVID-19. A meta-analysis conducted by Vardavas and Nikitara (2020) suggested that smoking increases the risk of developing severe COVID-19; however, another meta-analysis demonstrated that active smoking is not associated with enhanced risk in the progressing to severe COVID-19 (Lippi and Henry, 2020). There even are data indicate that smoking might have protective properties against SARS-CoV-2 (Farsalinos et al., 2020b; van Zyl-Smit et al., 2020). The challenge of studies focused on smoking and COVID-19 is that most hospitalized patients have underlying medical conditions such as hypertension, diabetes, cardiovascular disease and chronic obstructive pulmonary disease (COPD).

## Materials and Methods

### Study Design and Participants

Our multi-center study was performed in the four largest hospitals designated for COVID-19 patients in Wuhan. All hospitalized patients were confirmed COVID-19 positive according to the interim guidance from the World Health Organization and National Health Commission of China (World Health Organization, 2020), from February 01 to April 10, 2020. The severe illness was defined according to the New Coronavirus Pneumonia Prevention and Control Program (5th edition) issued by the National Health Commission of China (Supplementary Methods). The study was approved by the institutional ethics review board and the need for informed consent was waived.

### Data Collection

Clinical course, laboratory findings and outcomes were obtained and reviewed. The medical records were analyzed by three investigators independently. Information included demographic characteristics, symptoms, signs, underlying comorbid conditions and treatments were collected from the medical records. Laboratory data on the day of admission were collected. Smoking history was defined according to the WHO guidelines, including smoking for more than 6 months, or smoking more than 5 or more cigarettes per day (US Department of Health and Human Services, 2014); alcohol consumption was defined as an average of more than 7 standard drinks per week or more than 3 standard drinks per day (1 standard drink = 14 g of ethanol) (Burton and Sherons, 2018).

### Statistical Analysis

Categorical variables were described as frequency rates and percentages. Continuous variables were presented as mean ± standard deviation (SD) or mean, and interquartile range (IQR) as appropriately. We used the Mann–Whitney *U* test, x^2^ test, or Student’s *t*-test to compare differences between two different groups where appropriate. Univariable and multivariable logistic regression analyses were used to identify any association between serious illness and smoking history. Univariable and multivariable cox regression analyses were used to identify any association between death and smoking history. Variables with a *P*-value less than 0.05 at univariable analysis were subjected to multivariable analysis, and odds ratios with 95% confidence intervals (CIs) were reported. The areas under the receiver operating characteristic curve were used to evaluate the discriminative abilities of the disease severity status. *P*-values less than 0.05 were considered to indicate statistical significance. Statistical analyses were performed by using statistical software (SPSS version 26.0).

## Results

A total of 1547 subjects, including 802 male patients (51.8%) and 745 female patients (48.2%), were enrolled in the study. According to past medical history, the most common concurrent diseases were hypertension (27.34%), diabetes (12.93%) and cardiovascular disease (10.92%) (Supplementary Table 1). Among 1547 patients, there are 390 severe patients (25.21%), and 257 death patients (16.61%).

### Characteristics of COVID-19 Patients With and Without Smoking History

**FIGURE 1:**
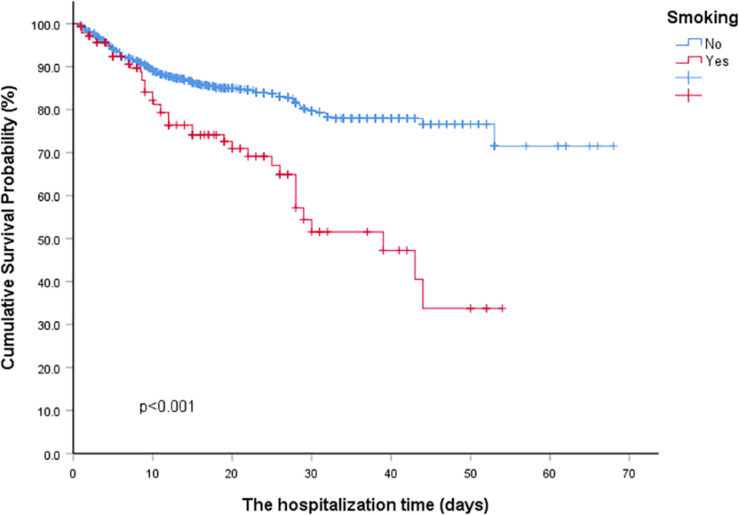
Kaplan-Meier survival curves for COVID-19 patients with and without Smoking history. *P < 0.01 for smoking group vs. non-smoking group.

Firstly, the population was divided into sub-groups according to whether they had a history of smoking. In the smoking group, the proportion of males was higher than that in the non-smoking group (87.59% vs. 48.15%, *p* < 0.001). There was no statistically significant difference in age, BMI, comorbidities and symptoms between the smoking group and the non-smoking group. However, in terms of biochemical indicators, the counts of lymphocyte and thrombocyte of the smoking group were lower than that of the non-smoking group, while C-reactive protein and AST were higher than that of the non-smoking group. The difference was statistically significant (all *p*-values < 0.05, Table 1). To further describe the effects of smoking, we conducted a Kaplan-Meier survival curves for COVID-19 patients. The results revealed that the smoking groups had greater deteriorated outcomes than the non-smoking groups (Figure 1).

**TABLE 1:** Characteristics of patients with COVID-19 with and without smoking history and alcohol consumption.

Characteristics	Total (*n* = 1547)	Smoking history		*P-*value^*a*^	Alcohol consumption		*P-*value^*b*^
		Yes (*n* = 145)	No (*n* = 1429)		Yes (*n* = 54)	No (*n* = 1493)	
Age (years)	57.31 ± 16.09	56.57 ± 15.89	57.39 ± 16.11	0.559	60.39 ± 10.68	57.17 ± 16.24	0.037
Sex				<0.001			<0.001
Female	745 (48.16%)	18 (12.41%)	727 (51.85%)		6 (11.11)	739 (49.56)	
Male	802 (51.84%)	127 (87.59%)	675 (48.15%)		48 (88.89)	752 (50.44)	
BMI	24.03 ± 3.22	23.96 ± 2.97	24.04 ± 3.25	0.866	24.12 ± 2.21	24.02 ± 3.27	0.820
Alcohol consumption	54 (3.5)	25 (16.7)	29 (2.1)	<0.001	25 (46.30)	125 (8.38)	<0.001
Comorbidities							
Hypertension	423 (27.34%)	43 (29.66%)	380 (27.10%)	0.512	21 (38.89)	400 (26.83)	0.051
Diabetes	200 (12.93%)	23 (15.86%)	177 (12.62%)	0.269	12 (22.22)	186 (12.47)	0.035
Cardiovascular disease	169 (10.92%)	17 (11.72%)	17 (11.72%)	0.746	8 (14.81)	160 (10.73)	0.344
Cerebrovascular disease	40 (2.59%)	4 (2.76%)	36 (2.57%)	0.890	8 (14.81)	160 (10.73)	0.548
Carcinoma	42 (2.71%)	4 (2.76%)	38 (2.71%)	0.973	2 (3.70)	40 (2.68)	0.650
Chronic lung disease	61 (3.94%)	9 (6.21%)	52 (3.71%)	0.141	2 (3.70)	57 (3.82)	0.964
Signs and symptoms							
Fever	487 (31.48%)	65 (44.83%)	489 (34.20%)	0.256	15 (27.78)	470 (31.52)	0.560
Cough	282 (18.23%)	24 (16.55%)	258 (18.40%)	0.583	5 (9.26)	276 (18.51)	0.083
Expectoration	42 (2.71%)	6 (4.14%)	36 (2.57%)	0.268	1 (1.85)	41 (2.75)	0.690
Myalgia	160 (10.34%)	21 (14.48%)	139 (9.91%)	0.085	6 (11.11)	154 (10.33)	0.853
Diarrhea	115 (7.43%)	12 (8.28%)	103 (7.21%)	0.485	4 (7.41%)	111 (7.43%)	0.872
Shortness of breath	115 (7.43%)	9 (6.21%)	106 (7.56%)	0.554	3 (5.56)	112 (7.51)	0.591
Laboratory Data							
WBC, × 10^9^/L	7.02 (2.08,16.78)	7.23 (2.58,16.78)	6.32 (2.08,13.20)	0.133	8.84 (3.88,13.23)	7.02 (2.08,16.78)	0.432
Neutrophil count, × 10^9^/L	4.85 (2.89, 5.2)	5.2 (3.29, 5.2)	4.76 (2.86, 5.2)	0.480	4.72 (3.2,5.55)	4.86 (2.88, 5.2)	0.827
Lymphocyte count, × 10^9^/L	1.32 (0.85, 1.47)	1.08 (0.68, 1.32)	1.32 (0.88, 1.5)	<0.001	1.06 (0.65,1.32)	1.32 (0.86,1.47)	0.015
Platelet count, × 10^9^/L	203.8 (155, 238)	179 (127, 209)	203.8 (160, 242)	<0.001	186.5 (142,241)	203.8 (156,238)	0.196
D-dimer, mg/L	1.26 (0.4, 14.7)	1.26 (0.4, 14.7)	1.26 (0.41, 14.7)	0.967	0.92 (0.49,10.29)	1.27 (0.39, 14.7)	0.684
C-reactive protein, mg/L	9.98 (1.4, 27.59)	23.9 (3.91, 34.3)	8.39 (1.29, 27.59)	0.002	7.18 (2.13,27.59)	10.4 (1.39,27.59)	0.977
ALT, U/L	27 (16, 35.78)	25.9 (17, 40)	27.25 (16, 35.78)	0.603	30.7 (20, 52.6)	27 (16, 35.78)	0.040
AST, U/L	26 (18, 40.05)	30 (20, 43)	25.3 (18, 40.05)	0.003	31 (21, 40.05)	26 (18, 40.05)	0.128
IL-6, pg/mL	28.67 (1.50,798.00)	45.93 (1.50,798.0)	6.52 (1.50, 496.70)	0.503	32.02 (1.50, 798.0)	6.52 (1.50, 374.12)	0.514
Procalcitonin, ng/mL	0.43 (0.02, 57.17)	0.56 (0.05, 57.17)	0.12 (0.02, 28.45)	0.069	0.38 (0.02, 40.08)	0.58 (0.02, 57.17)	0.103

*Data are presented as mean ± SD or *n* (%). Laboratory data on the day of admission were collected. ^*a*^*P-*values indicate differences between COVID-19 patient with and without smoking history; ^*b*^COVID-19 patient with and without Alcohol consumption; *P* < 0.05 was considered statistically significant. BMI, body mass index; WBC, white blood cell count; ALT, alanine transaminase; AST, aspartate transaminase.*

### Characteristics of COVID-19 Patients With and Without Alcohol Consumption

Within the same population, two groups were divided according to whether they had a history of alcohol consumption. The proportion of males in the alcohol group was higher than that in the non-alcohol group (88.89% vs. 50.44%, *p* < 0.001). There was no statistically significant difference in age and BMI. Concerning comorbidities and symptoms upon admission, COVID-19 patients with and without alcohol consumption were similar except for a higher proportion of diabetes (22.22% vs. 12.47%, *p* = 0.035). In terms of laboratory indicators, the counts of lymphocyte from the alcohol group were lower than that of the non-alcohol group, while ALT was higher than that of the non-alcohol group. The difference was statistically significant (*p* < 0.05, Table 1). The Odds Ratio (OR) for the effect of CS on severe illness was 1.910 (95% CI = 1.203–3.033, *p* = 0.001), indicating that the risk of severe illness in patients with previous CS history was 1.91 times higher than that in non-smokers.

### Risk Factors Associated With Severe Illness and Mortality

However, in both univariable and multivariable analyses, alcohol consumption did not reveal any significant effect on developing severe illness in COVID-19 patients (Table 2). Among them, CS had a Hazard Ratios (HR) = 1.825 (95% CI = 1.275-2.613, *p* = 0.001), indicating that the risk of death in smokers was 1.825 times higher than that in non-smokers. Also, lymphocyte count, C-reactive protein, and ALT were risk factors for death. However, alcohol consumption did not show a significant effect on death rates of COVID-19 patients in both univariable and multivariable analyses (Table 3). In this study, we analyzed the association between alcohol consumption, CS, and the risk of COVID-19. Our findings indicated that COVID-19 patients with a history of CS tend to have more severe outcomes than non-smoking patients. However, alcohol consumption did not reveal significant effects on neither development of severe illness nor death rates in COVID-19 patients.

**TABLE 2:** Risk factors associated with severe illness by logistic regression.

	Univariable analysis			Multivariable analysis		

	cOR	95% CI	*p*	aOR	95% CI	*P*
Age	1.052	1.043–1.062	<0.001	1.026	1.015–1.038	<0.001
Gender	1.936	1.528–2.453	<0.001			
BMI	0.992	0.923–1.084	0.992			
Smoking	1.955	1.375–2.779	<0.001	1.910	1.203–3.033	0.001
Alcohol consumption	1.639	0.926–2.900	0.090			
Diabetes	4.313	3.168–5.871	<0.001	3.713	2.432–5.668	<0.001
Hypertension	3.151	2.468–4.024	<0.001	1.644	1.154–2.342	0.006
Chronic lung disease	3.012	1.784–5.087	<0.001	3.114	1.370–7.078	0.007
Cerebrovascular disease	4.256	2.211–8.190	<0.001			
Cardiovascular disease	2.719	1.956–3.779	<0.001			
Carcinoma	3.393	1.831–6.287	0.001			
Lymphocyte count	0.993	0.991–0.994	<0.001	1.192	1.123–1.264	<0.001
Neutrophil count	1.379	1.316–1.446	<0.001			
C-reactive protein	1.036	1.030–1.041	<0.001	1.021	1.015–1.026	<0.001
ALT	1.010	1.006–1.014	<0.001			
AST	1.027	1.021–1.032	<0.001	1.011	1.005–1.017	<0.001
D-dimer	1.000	0.999–1.001	0.964			

*Data in parentheses are 95% confidence intervals. *P* < 0.05 was considered statistically significant; ALT, alanine transaminase; AST, aspartate transaminase. In the univariate model, risk ratios were not adjusted for any confounders. In multivariate model, risk ratios were additionally adjusted for confounders with *p* < 0.05 in the univariate model, and these independent variables are corrected for each other, thus obtaining the corrected partial regression coefficient (aOR/aHR).*

**TABLE 3:** Risk factors associated with mortality by cox regression.

	Univariable analysis			Multivariable analysis		

	cHR	95% CI	*p*	aHR	95% CI	*P*
Age	1.054	1.044–1.064	<0.001	1.031	1.019–1.041	<0.001
Gender	2.216	1.638–2.759	<0.001			
BMI	0.984	0.912–1.061	0.671			
Smoking	2.265	1.627–3.153	<0.001	1.825	1.275–2.613	0.001
Alcohol consumption	1.200	0.656–2.196	0.554			
Diabetes	2.280	1.721–3.019	<0.001	1.453	1.063–1.985	0.019
Hypertension	2.365	1.852–3.018	<0.001			
Chronic lung disease	2.899	1.918–4.38	<0.001			
Cerebrovascular disease	3.464	2.194–5.47	<0.001			
Cardiovascular disease	2.295	1.716–3.07	<0.001	1.485	1.078–2.045	0.015
Carcinoma	2.435	1.445–4.102	0.001			
Neutrophil count	1.101	1.091–1.111	<0.001	1.071	1.054–1.089	<0.001
Lymphocyte count	0.309	0.238–0.400	<0.001			
C-reactive protein	1.008	1.007–1.009	<0.001	1.007	1.006–1.009	<0.001
ALT	1.002	1.001–1.003	<0.001	1.001	1.000–1.002	0.010
AST	1.001	1.001–1.001	<0.001			
D-dimer	1.001	1.000–1.001	0.263			

*Data in parentheses are 95% confidence intervals. *P* < 0.05 was considered statistically significant; ALT, alanine transaminase; AST, aspartate transaminase. In the univariate model, risk ratios were not adjusted for any confounders. In multivariate model, risk ratios were additionally adjusted for confounders with *p* < 0.05 in the univariate model, and these independent variables are corrected for each other, thus obtaining the corrected partial regression coefficient (aOR/aHR).*

## Discussion

Alcohol is the most commonly abused drug in the world. It affects almost every organ of our body. At this time, patients with a history of alcohol consumption did not develop more severe outcomes compared to patients without. That might be because the time we collect our data is still in the early stage of the pandemic. With the SARS-CoV-2 rapidly spreading, governments across the world have issued stay-at-home and face covering orders, which resulted in trillions of people being isolated from each other for long periods of time. Combining with the stress of rising unemployment, excessive use of alcohol consumption becomes a public health crisis (Clay and Parker, 2020). Several meta-analyses suggested that there is a significant association between COVID-19 and CS; however, they also admitted their analyses were limited by sample size and number of primary researches (Farsalinos et al., 2020a). In our study, the counts of lymphocyte of the smoking group were lower than that of the non-smoking group, which may contribute to the deteriorated outcomes. Immunosuppression caused by smoking inhibits the effective activation of T cells, which also inhibit B cells to proliferate and produce antibodies, thus making humoral immunity incapable (McBride et al., 2003). C-reactive protein is positively correlated with smoking. Increased inflammatory in COVID-19 patients with smoking history could contribute to the worse outcome in this subpopulation (Chen et al., 2020). Studies have shown that smoking may increase multiple enzymes in the human liver. Cirrhosis of the liver has a recognized immune dysfunction status that includes immunodeficiency and systemic inflammation, making it reasonable for those patients to be more susceptible to SARS-CoV-2 infection (Velarde-Ruiz Velasco et al., 2020). In this study, the data we collected containing a total of 1,547 patients with 150 smokers, which will provide a better understanding of the effects of CS in COVID-19. Based on our analysis, COVID-19 patients with a history of CS had more severe outcomes when compared to the population without a CS history.

In conclusion, CS is a risk factor for developing severe illness and increasing mortality during the SARS-CoV-2 infection. We believe that our findings will provide a better understanding on the effects of alcohol intake and CS exposure in COVID-19 patients.

## Author Contributions

ML designed the research. MD and ZC were in charge of data collection and manuscript writing. All authors contributed to the article and approved the submitted version.

## Conflict of Interest

The authors declare that the research was conducted in the absence of any commercial or financial relationships that could be construed as a potential conflict of interest.
