# Supplementary material for: Influence of Cigarettes and Alcohol on the Severity and Death of COVID-19: A Multicenter Retrospective Study in Wuhan, China
Source: Front Physiol. 2020 Dec 9;11:588553. doi: 10.3389/fphys.2020.588553 (PMC7756110; doi:10.3389/fphys.2020.588553)
Supplement: Supplementary file 1 [file Table_1.DOC]

**Supplementary Methods**

The 5th edition 2019 Novel Coronavirus Disease (COVID-19) Diagnostic criteria (published by the National Health Commission in China on 5 Feb 2020)

1. **Diagnostic Criteria**

***Other provinces except Hubei province***

1. Suspected cases by clinical criteria

Comprehensive screening of the following epidemiological information and clinical manifestations:

1) Epidemiological information

a) Cases reporting a history of travel to or reside in Wuhan city and surrounding areas, or other communities with reported cases within 14 days prior to onset of illness.

b) Cases have been exposed to patients with COVID-19 (confirmed by laboratory diagnosis of SARS-CoV-2 RNA detection) within 14 days prior to onset of illness.

c) Cases have been exposed to patients with fever or respiratory symptoms from Wuhan city and surrounding areas, or other communities with reported cases within 14 days prior to onset of illness.

d) Has contact history with a cluster.

2) Clinical manifestations

a) Fever and/or respiratory symptoms

b) Computed tomography (CT) changes for viral pneumonia

c) Normal or below normal absolute leukocyte count, or low absolute lymphocyte count below normal range in the early stages of the disease.

2. Diagnosis of COVID-19 is made if meeting one of the following:

1) cases report any one of above epidemiological information and harbor any two of the clinical manifestations;

2) cases without the above epidemiological information, but harbor all of the above clinical manifestations.

***Hubei province***

1.Suspected cases.

Comprehensively analyze combinations of the following epidemiological history and clinical presentations:

1) Eidemiological history

a) Within 14 days prior to onset, had history of travel or residence in Wuhan or surrounding regions, or other communities reporting cases.

b) Within 14 days prior to symptom onset, having had contact with patients infected with 2019- nCoV (positive nucleic acid test).

c) Within 14 days prior to onset, had contact with patients who had a fever or respiratory tract symptoms that had come from Wuhan, its surrounding regions, or other communities reporting cases.

d) Clustered onset (Within a span of 2 weeks, 2 or more cases with fever and/or respiratory symptoms appear in a small area, such as a family, an office, or a school class).

2) Clinical presentations

a) Fever and/or respiratory tract symptoms;

b) Having the imaging features of novel coronavirus pneumonia discussed above;

c) During the early stages of the disease, white blood cell count is normal or reduced, while the lymphocyte count is normal or reduced;

Diagnosis of COVID-19 is made if meeting one of the following:

1)any of the epidemiologic history items, and any 2 of the clinical presentions are met;

2)there is no clear epidemiological history, and at least 3 of the clinical presentations are met.

2. Confirmed cases

1) Clinically diagnosed cases

CT chest changes that are consistent with atypical viral pneumonia.

2) Laboratory confirmed cases

a) Respiratory tract or blood specimen test positive for SARS-CoV2 by real-time reverse transcriptase polymerase chain reaction (RT-PCR) assay.

b) The sequence of the virus is highly homologues to that of SARS-CoV-2.

1. **Clinical definitions of severe symptoms**

Adults who meet any one of the following:
1.Shortness of breath, RR>30 breaths/minute;

2.Oxygen saturation<93% at rest;

3.Arterial oxygen partial pressure (PaO2)/ fraction of inspired oxygen (FiO2)<300mmHg(1mmHg=0.133kPa);

4.The patient should be managed as a severe case if lung imaging shows a substantial progression of lesions (greater than 50%) within 24-48 hours.
